# Editorial: The role of nutritional supplements and oral complementary or alternative medicine supplements for the management of chronic conditions in children

**DOI:** 10.3389/fped.2026.1803405

**Published:** 2026-03-04

**Authors:** Angharad Vernon-Roberts, Andrew S. Day

**Affiliations:** Department of Paediatrics and Child Health, University of Otago Christchurch, Christchurch, New Zealand

**Keywords:** CAM therapies, chronic conditions, non-disclosure, nutrition, pediatric, supplementation

Children with chronic conditions may experience a wide range of somatic and psychosomatic symptoms resulting from their illness (1, 2). Allopathic treatments may provide symptom resolution or relief, but clinicians are increasingly incorporating non-prescription supplementation into treatment regimens for their patients (3). Research on the role that nutritional and complementary or alternative medicine (CAM) supplementation may have in the treatment of chronic disease is becoming more prevalent with many promising results arising. The use, efficacy, and safety concerns of non-prescription supplementation for children with chronic conditions are important topics to address. It is only via increased visibility of research on CAM and nutritional supplementation, either as stand-alone therapy or in conjunction with prescribed medications, that clinicians can provide holistic, fully informed care for children with chronic conditions.

Establishing the extent of CAM or nutritional supplementation use, and factors associated with this practice, may help inform education materials and promote awareness of potential safety concerns for clinicians and carers (4). The use of supplementation for children is reported to vary between 4% and 100% for short-term use, and 48 to 90% for lifetime prevalence (5). The safety of non-disclosure of CAM use is high, reported at 67% in one meta-analysis (6), and in individual studies among children from 60 to 99% non-disclosure to the child's medical team (7). Known interactions between prescribed medication and non-prescription supplements pose significant safety risks. In addition the reasons for non-disclosure of supplementation use are important to establish, with the most common reason being not being asked by doctors or thinking CAM use wasn't important to mention (6, 8). In this Research Topic Tekle et al. assessed traditional medicine (TM) usage among a cohort of children attending a tertiary care hospital. The prevalence of TM use was high with 86.5% of parents reporting having given their child TM within the last twelve months, and of those using it 27% had given oral TM, 30% topical, and 38% inhaled. The main reasons given for TM use were the belief that modern medicines cannot cure some diseases, and that TM is effective when chosen and used correctly. In this report, only 13% of parents using TM for their child had disclosed this to healthcare professionals, the main reason being that they were not asked. However, 95% of the respondents stated they would discontinue use if their child got sicker after using it, and 81% would stop if their doctor asked them to discontinue use. The most significant factor associated with TM use for their child was having used TM themselves, and parents having a “good” perception of TM efficacy. This study adds further weight to growing evidence of high levels of CAM use among children with an associated high non-disclosure rate that poses potential safety concerns.

CAM or nutritional supplementation may be used as preventative treatment for childhood conditions, with specific health challenges being evident in regions around the world. When considering CAM as a treatment modality the synthesis of available evidence helps to inform clinical decision-making. Children in Sub-Saharan Africa are at risk of iron deﬁciency anemia and undernutrition secondary to soil-transmitted helminths and infections such as malaria (9). Kedir et al. report a meta-analysis of the evidence relating to weekly iron-folic acid supplementation (WIFAS) and its effect on the nutrition status, helminth re-infection, and malaria infections of school-age children and adolescents in Sub-Saharan Africa. In their synthesis of eleven included studies, they found that WIFAS decreased the risk of reinfection of the parasitic disease schistosomiasis by 21% among adolescents, but it did not reduce the risk of malaria or Lumbricoides reinfection. In addition, WIFAS did not improve height or height for age Z-scores in school-age children. The results of this study may be used to ensure appropriate use of WIFAS supplementation for children vulnerable to certain parasitic infections in Sub-Saharan Africa.

Vitamin supplementation has been used for children with various chronic conditions and having adequate micronutrient levels are considered important for overall child health (10). Specifically, vitamin B is considered vital for cellular function (11) and vitamin D for phosphate and calcium metabolism and essential for bone health (12). However, evidence is lacking on the wider benefits of these vitamins for specific health conditions (12, 13). A study by Kovalchuk et al. reports in this Research Topic on the effectiveness of vitamin B6, B9, B12, and D3 supplementation upon reducing symptoms and frequency of syncope, autonomic nervous system function, and quality of life (QOL) in children following an episode of vasovagal syncope (VVS). The study reported that adolescents with VVS who took vitamin B and D supplements consistently for three months experienced a reduced frequency of syncopal episodes and symptoms. The children also had decreased levels of homocysteine, which are known to be associated with autonomic function. Heart rate variability and cardiac autonomic function were both reported to have improved following supplementation, as did blood pressure indicators. Additionally, during the three-month treatment interval children showed improvements in QOL within the domains of physical, emotional, and school functioning when self-reported by the children and proxy-reported by their parents. Parents themselves experienced higher levels of QOL within the family impact domain with enhanced physical, emotional, social, and cognitive functioning, alongside improved communication, reduced worry levels, improved daily activities, and better family relationships. This research shows promise for many clinical and psychosocial outcomes for children with VVS and their carers consequent to the use of a commonly available supplement. Careful consideration should be given to dissemination of results such as this to ensure appropriate use and safety.

The consideration of novel CAM therapies as adjunctive treatments for common childhood conditions is an important field of research, particularly for conditions with high prevalence. Approximately 10% of children have asthma, with rates ranging from 8 to 23% throughout different regions of the world (14). While this represents a significant burden on children, families, and healthcare systems little research has explored the use of adjunctive therapies to complement standard prescription drugs (15). In this Research Topic Luo et al. explore the use of Xiao-er Kechuanling (XKL) as an adjunctive treatment alongside short-acting beta2-agonists (SABA) and leukotriene receptor antagonists (LTRA) in children with asthma. XKL is a traditional Chinese medicine that has demonstrated anti-inﬂammatory and bronchodilatory effects (16). The study compared the use of triple therapy using XKL, the SABA terbutaline, and LTRA montelukast against dual therapy with just terbutaline and montelukast, among a cohort of children with uncontrolled asthma. After both three and six-months of treatment, more children on the triple therapy achieved improvements in asthma control, greater reductions in the cytokine Transforming growth factor *β* and matrix metalloproteinases-9/tissue inhibitor metalloproteinase-1 that indicate airway inflammation and bronchial injury. There was, however, no difference between the groups for frequency of exacerbations requiring hospitalization. The triple therapy group demonstrated greater improvements in spirometry markers (FEV1/FVC) after three months of treatment compared to the dual therapy group, with this difference persisting and increasing by month six. The use of CAM as adjunctive therapy for children with asthma in this study shows great promise for many clinical indices and contributes to a small but growing body of evidence on the use of adjunctive therapies for this condition.

In presenting this Research Topic it is hoped that increasing awareness of the association between CAM/nutritional supplementation and positive outcomes for children with chronic conditions may encourage further research. With rates of use of CAM being high among the pediatric population it is important to make informed clinical decisions based on available evidence. This work also highlights that ongoing non-disclosure of CAM/nutritional supplementation needs to be addressed as an urgent safety issue in the healthcare sphere.
